# High-Density Microwell Chip for Culture and Analysis of Stem Cells

**DOI:** 10.1371/journal.pone.0006997

**Published:** 2009-09-14

**Authors:** Sara Lindström, Malin Eriksson, Tandis Vazin, Julia Sandberg, Joakim Lundeberg, Jonas Frisén, Helene Andersson-Svahn

**Affiliations:** 1 Division of Nanobiotechnology, AlbaNova University Center, Royal Institute of Technology, Stockholm, Sweden; 2 Department of Cell and Molecular Biology, Karolinska Institute, Stockholm, Sweden; 3 Division of Gene Technology, AlbaNova University Center, Royal Institute of Technology, Stockholm, Sweden; 4 Cellular Neurobiology Research Branch, National Institute on Drug Abuse, National Institutes of Health, Department of Health and Human Services, Baltimore, Maryland, United States of America; City of Hope Medical Center, United States of America

## Abstract

With recent findings on the role of reprogramming factors on stem cells, *in vitro* screening assays for studying (de)-differentiation is of great interest. We developed a miniaturized stem cell screening chip that is easily accessible and provides means of rapidly studying thousands of individual stem/progenitor cell samples, using low reagent volumes. For example, screening of 700,000 substances would take less than two days, using this platform combined with a conventional bio-imaging system. The microwell chip has standard slide format and consists of 672 wells in total. Each well holds 500 nl, a volume small enough to drastically decrease reagent costs but large enough to allow utilization of standard laboratory equipment. Results presented here include weeklong culturing and differentiation assays of mouse embryonic stem cells, mouse adult neural stem cells, and human embryonic stem cells. The possibility to either maintain the cells as stem/progenitor cells or to study cell differentiation of stem/progenitor cells over time is demonstrated. Clonality is critical for stem cell research, and was accomplished in the microwell chips by isolation and clonal analysis of single mouse embryonic stem cells using flow cytometric cell-sorting. Protocols for practical handling of the microwell chips are presented, describing a rapid and user-friendly method for the simultaneous study of thousands of stem cell cultures in small microwells. This microwell chip has high potential for a wide range of applications, for example directed differentiation assays and screening of reprogramming factors, opening up considerable opportunities in the stem cell field.

## Introduction

Stem cells have been studied for nearly 50 years and while our understanding of them has increased immensely, major questions such as their molecular identity, level of plasticity, and role in pathological conditions and aging, still remain to be answered. Stem cells are identified by their functional characteristics; multipotency and self-renewing capability. In the present study, mouse- and human embryonic stem (ES) cells and adult neural stem cells from the mouse forebrain were used. Adult neural stem cells can be maintained in culture for several passages and display multipotency by generating neurons, astrocytes, and oligodendrocytes upon differentiation.

Mammalian embryonic and adult neural stem cells have been successfully isolated and maintained *in vitro* [1], [2]. Culture conditions vary and ES cells are often cultured adherently on gelatin coatings or fibroblast feeder cells, whereas adult neural stem cells are maintained as free-floating sphere-like clusters, called neurosphere (NS) cultures. Furthermore, maintenance of pluri- or multipotency in different stem cell populations depends on various factors such as leukemia inhibitory factor (LIF) [3], [4], epidermal growth factor (EGF), and basic fibroblast growth factor (bFGF) [2], [5], [6]. New factors are constantly connected to stem cells regulating their maintenance or differentiation, *e.g.* growth factors, epigenetic modifiers, neurotransmitters, and extracellular matrix proteins.

Extensive studies have been aimed at finding specific combinations of factors for directed differentiation, maintenance of stem cell populations, and for the reprogramming of mature cells into induced-pluripotent cells [7]–[9]. Such screens may be limited by the cost of the compounds to be analyzed, whereby true low-volume-assays would enable greater numbers of parallel experiments. As the understanding and knowledge on stem cells continue to increase, demands on the experimental tools and methods follow the same direction. Platforms that enable high throughput analysis of individual stem/progenitor cells can provide key insights into the molecular regulation of stem cell maintenance, differentiation, and the identification of stem cells [10]. Microtiter-, microwell-, or multi-well plates have long been used in research, as tools for increasing assay throughput and reduce cost. These are typically made of polystyrene and provide from 6 to 3456 individual wells, in which the samples are mixed with reagents, agitated and incubated, either manually or with automated handling equipment. The volumes involved in conventional microtiter formats range from 16 000 µl (6 well plate, Greiner), 400 µl (96 well plate, Nunc), down to 2.6 µl (3456 well plate, Aurora). The 96- or 384-well plates are the most frequently used formats. However, the analysis of individual stem cells within such formats is far from optimal. To better control the cellular microenvironment, further miniaturization by microsystem technologies [11], [12] can provide supplementary culturing- and analysis platforms for stem cell research [13]–[18],. The aim of this work was to provide a miniaturized *in vitro* assay for multi-parallel culturing and analysis of single stem cells, by combining chip-based methods with conventional microwell plate technologies.

The significant single-cell variability within a cell population is widely studied, for example in the isolation of stem cells and their clones. In order to better understand cell heterogeneity and detect rare interesting cells in a large population, methods that are capable of rapidly analyzing single cells are desired. Cell chips consisting of many small wells are common approaches for studying single cells, where many of the methods involve uncontrolled, random settling of cells into microwells [19]–[21]. Surface micropatterning [22]–[24] and microfluidics [25]–[30] are two other promising ways to investigate stem cells, as compared to conventional bulk analysis. Examples of stem cell studies using microdevices are culturing of homogeneously sized embryoid bodies (EBs) [31], [32], 3-D microwell culture of human embryonic stem (ES) cells [33], and neurosphere (NS) cultures. For *in vitro* neurosphere formation assays; clonality is crucial and investigations on movement-induced aggregation of clonal spheres and possible solutions thereof have been investigated earlier in bulk samples as well as in miniaturized systems [34]–[36]. General obstacles of existing cell chips are controlled single-cell seeding, clonality assurance, long-term cell culturing and cell maintenance, *i.e.* to ensure that the exact same cell is being studied over time.

We developed a method where thousands of single, or a controlled number of, stem cells and their neural differentiation can be studied individually on a microwell chip with high density of wells. The chip has previously been used for heterogeneity analysis of single carcinoma cells and their clonal expansion [37]. The slide-formed chip (26×76 mm) consists of 672 microwells where each well holds 500 nl; a volume that is small enough to dramatically decrease reagent costs but large enough to allow utilization of standard laboratory equipment. Controlled cell seeding and culturing is achieved by the unique compatibility of the microwell chip and conventional flow cytometric cell-sorting instruments, facilitating clonal assays. Further strengths are the i) relatively large size of the microwells (650×650 µm) for long-term culturing, ii) reversibly sealed wells which hinder cell migration and evaporation, iii) good optical properties and compatibility with imaging and screening systems, and iv) user-friendly handling of the chip. The microwell chip presented here gives the opportunity to monitor and manipulate stem cells in a new way, enabling individual treatment, clonal assays, and maintenance and differentiation studies of stem cells in high throughput.

## Results

### Chip properties and liquid handling

**Figure 1:**
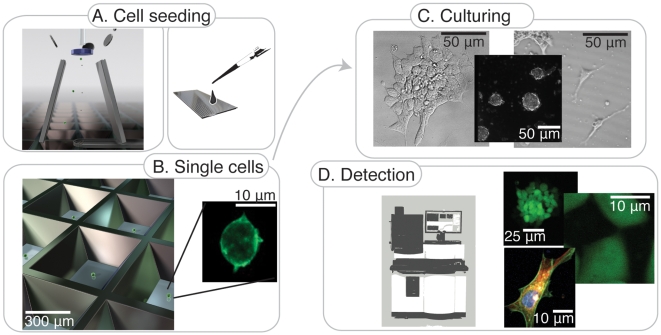
Schematic overview of screening method. (A): Cells are seeded into the microwells, either by automatic instrumentation such as flow cytometric cell-sorting (left), or manually by limited dilution (right). (B): One cell per well opens up for heterogeneity screenings, clonal assays, among other applications. Zoom in on a single cell, fixed and labeled directly after cell seeding. (C): Cell analysis, weeklong culturing and differentiation studies can be performed. Culture medium change can be performed, by rinsing the chip with fresh medium. (D): The entire chip can be screened in a rapid manner using conventional automated imaging systems, detecting cells and clones in the 672 individual wells simultaneously.

A general method using a miniaturized microwell chip for stem cell studies and screening applications is schematically described in Figure 1. Random cell seeding by manual pipetting can be utilized (Fig. 1A, right), either by addressing individual wells or by addressing all wells simultaneously. In the latter, a volume of 800 µl of cell suspension is dispensed onto the chip, spread over the chip area and the cells are randomly settled in the wells by adding a cell culture top membrane. An alternative seeding option is controlled automated cell seeding in accordance with the well-known procedure of clonal assays using flow cytometric cell-sorting [38] (Fig. 1A, left). The compatibility of the microwell chip presented here and flow cytometric cell-seeding, is unique for this particular chip in contrast to other stem cell chips and greatly increases the probability of achieving true clonality, as described in detail earlier [37]. Each cell or clone has its own microchamber without contact and risk of aggregation with neighboring cells or clones (Fig. 1B). The cells can be analyzed instantly or subjected to short- or long-term culturing (Fig. 1C). Rinsing the chip with fresh culture medium can easily perform change of medium. Live-cell imaging or endpoint analysis and detection can be carried out using automated imaging systems or standard microscopy (Fig. 1D).

**Figure 2:**
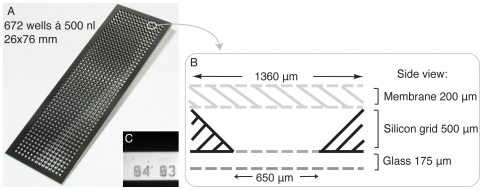
Microwell chip. (A): Photograph of a microwell chip holding 672 microwells on a slide format of 26×76 mm. (B): Schematic drawing of one well with a volume of 500 nl, constructed as a sandwich with a glass bottom bonded to a silicon grid, creating wells. For cell culturing, a reversibly added silicone membrane is used. (C): Photograph of a well number, situated in between all wells, describing its row and column-position to enable tracking of wells for repeated imaging.

The slide-formed chip (26×76 mm) consists of 672 microwells where each well holds a total volume of 500 nl (Fig. 2A). A simple and robust protocol for dispensation and aspiration of liquids in the many wells using a regular pipette was developed. Due to the shallow wells and sloped walls of the microwells, liquid could be exchanged in less than 1 min by rinsing on top of the chip. An absorbing paper tissue was employed to empty the microwells. The flat bottom- and top surfaces together with the cover slip thickness (175 µm) of the glass bottom (Fig. 2B) made the chip well suited for imaging. A thin (200 µm) cell culturing silicone membrane was used for high quality live cell imaging (Fig. 2B). The microfabricated numbering of each well enabled cell- and well tracking on chip (Fig. 2C). For automated high throughput experiments, there are commercially available robotics that can dispense liquid onto the entire chip in a couple of minutes. Hence, the microwell chip can be used both by manual and automated liquid handling.

### Stem cell culturing and neuronal differentiation on chip

**Figure 3:**
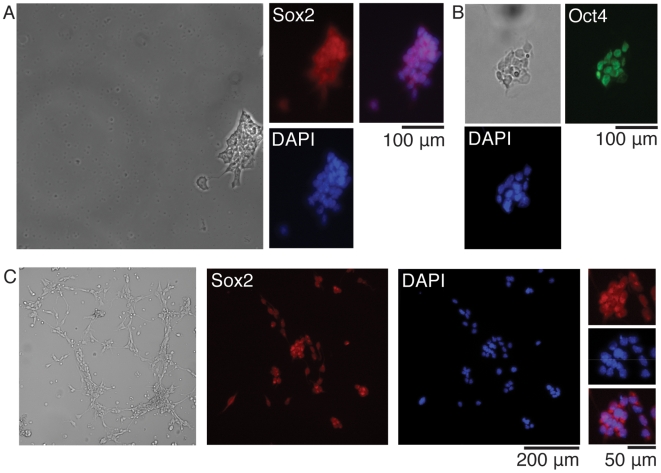
Pluripotency on chip. (A, B): Mouse ES cells three days after plating on chip. Cells are immunoreactive to the pluripotency markers Sox2 (red) (A) and Oct4 (green) (B) and counterstained with the nuclear stain DAPI (blue). (A): Micrograph of an entire well shown in bright field (left). Close up on the colony, showing Sox2 positive ES cells, DAPI staining, and overlay of Sox2 and DAPI. (B): Close up on a colony in bright field, along with Oct4 positive ES cells, and DAPI staining. (C): Mouse adult neural stem cells three days after plating on chip. Live-cell micrograph of an entire well shown in bright-field (left). Whole-well images on Sox2 and DAPI, as well as close ups on each staining along with overlay. All micrographs were obtained using a 10× objective.

Microwell chip culture of murine ES, murine adult forebrain neural stem cells, and human ES cells was possible both for maintenance of cells in a pluripotent state, or for differentiation into neuronal fates. Control experiments were simultaneously performed in 96-well plates, throughout the study. The pluripotent state was demonstrated by fixation and labeling of the cells with the pluripotency markers Sox2 and Oct4, and counterstained with the nuclear stain DAPI, three days after cell seeding on chip (Fig. 3). Cells were immunoreactive to the pluripotency markers, demonstrating the ability to maintain pluripotency of the investigated stem cells on chip.

**Figure 4:**
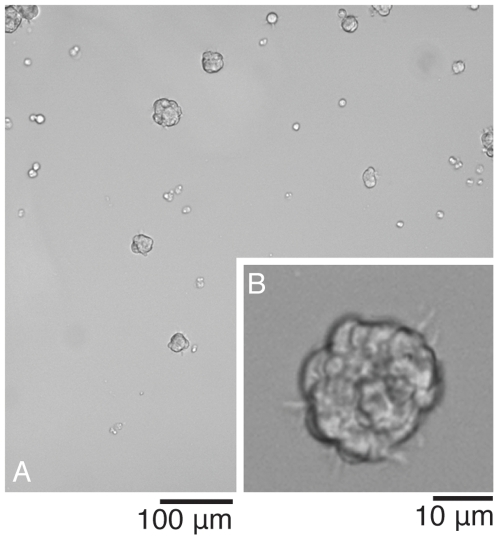
Neurospheres. Mouse adult neural stem cells in a single-cell suspension were seeded into an uncoated chip. (A): Live cell image of an entire well showing early neurosphere formation, three days past passage. (B): Close up on a single sphere, four days past passage. Micrographs were obtained using a 10× objective.

Furthermore, the microwell chip proved to be suitable for culturing adult neural stem cells in the form of neurospheres, an assay often used to demonstrate self-renewal *in vitro*. Single adult neural stem cells were seeded followed by monitoring over a period of four days, resulting in spheres in the range of 8–64 cells (Fig. 4). Fresh medium was added after three days to allow continued cell expansion (Fig. 4B). Spheres with a diameter of 500 µm should be possible to grow on chip, thereafter limited by the size of the well. Problems of movement-induced aggregation [35], [36] and doubts of whether a sphere is clonally derived can be avoided by seeding one cell per well. Walls making up the wells prevent neighboring spheres from contact, *i.e.* the risk of sphere fusion and/or cell exchange between spheres is eliminated and true clonality of spheres at the end of experiment can be obtained. Even in experiments with 10–20 cells per well at start, the small size of each well makes it easy to keep track of each individual sphere and its expansion (Fig. 4A). Additionally, since the wells are too small to create any significant movements of medium and cells within the wells, movement-induced aggregation should be greatly reduced.

**Figure 5:**
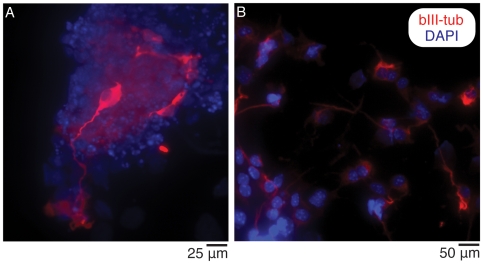
Neural differentiation of mouse stem cells. (A): Differentiation of ES cells under neuronal permissive conditions, nine days after plating on chip. (B): Differentiation of adult neural stem cells. Dissociated NS cells were plated and differentiated under neuronal permissive conditions for nine days on chip. Cells are immunoreactive to the neuronal marker βIII-tubulin (red) and counterstained with the nuclear stain DAPI (blue). Micrographs were obtained using a 10× objective.

**Figure 6:**
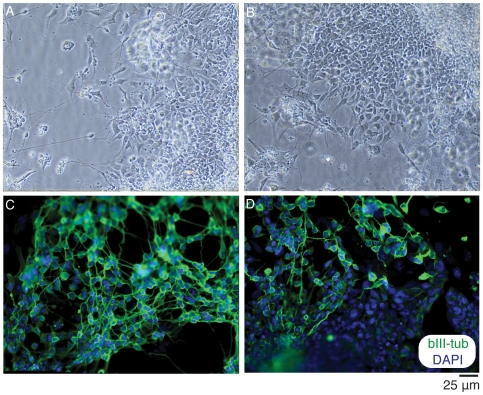
Culture and neural differentiation of EBs derived from BG01 and BG01V2 hESC. Phase contrast images of differentiating EBs derived from (A) BG01 and (B) BG01V2 illustrating that human ES cells survive well and are capable of undergoing differentiation after three days of culture in the microwells. Neuronal differentiation was confirmed by expression of the neuronal marker βIII-tubulin (green) in EBs generated from (C) BG01 and (D) BG01V2 differentiated for nine days. At this time, expression of the pluripotency marker Oct3/4 was completely lost. The cell cultures were counterstained with the nuclear stain DAPI (blue). Micrographs were obtained using a 10× or 20× objective.

The microwell chip was confirmed useful for differentiation studies of neural stem cells. The chip was swiftly coated with gelatin, poly-L-lysine, or poly-L-ornithine/laminin followed by seeding of mouse ES cells, adult neural stem cells, or human ES cells respectively. The mouse ES cells (Fig. 5) and human ES cells (Fig. 6) were maintained under differentiation conditions for 9 days, followed by fixation and labeling with the neuronal marker βIII-tubulin and counterstained with DAPI. Cells that were immunoreactive to the neuronal marker were found on chip, demonstrating its potential use in differentiation assays. Based on previous cell culturing on chip, it is possible to culture cells for at least 2–3 weeks on the microwell chip [37] but this depends on the particular cell type being cultured. Since culture medium can be exchanged, the main limitation for long-term cell culturing is confluency.

### Clonal assays on chip

**Figure 7:**
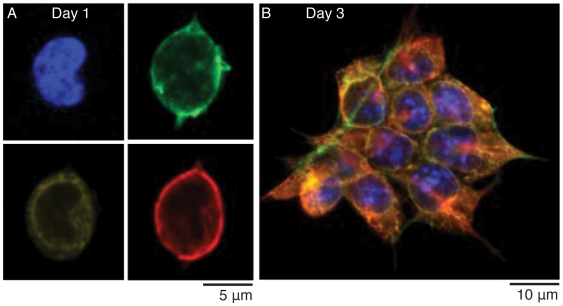
Clonal assay of mouse ES cells. Single cells were seeded into individual wells in microwell chips using flow cytometric cell-sorting, and cultured for (A) one and (B) three days. At time for analysis, the cells were visualized by staining for filamin (green), calreticulin (yellow), tubulin (red), and nucleus (blue, DAPI). (B): A clone shown with overlay of all channels. Micrographs are close ups on the cell/clone and were obtained using a 40×W objective.

As an alternative to 96-well plates, a relatively large format for small clones, the microwell chip offers a suitable well size for single-cell and clonal analysis. A unique property of the chip is its compatibility with conventional flow cytometric cell-sorting, as compared to other cell chips. The center-to-center distance of the microwells on the chip was designed to match the smallest movement of plate holders of conventional flow cytometer instruments. As a proof-of-principle, fluorescence activated cell sorting (FACS) was applied for seeding single murine ES cells on the chip, using the standard plate sort fitting of the instrument, followed by clonal analysis. Two chips were seeded in parallel whereby one was fixed and analyzed on day 1 (Fig. 7A) and the other chip was kept until day 3 (Fig. 7B) before analysis. To visualize the cells, the cultures were stained with antibodies against filamin (green), calreticulin (yellow), and tubulin (red). Nuclei were stained with DAPI (blue). Cultured cells, forming clones, indicate the potential use of the microwell chip for high-throughput clonal assays.

## Discussion

We describe a novel miniaturized multiwell chip for stem cell culturing and analysis as a promising tool in factor screening and stem cell research in general. The high-density microwell chip is easy to use, provides rapid screening, and should therefore be easily adapted to a wide range of research applications. The miniaturized format is in particular suitable for single-cell and clonal analysis, and provides more information of heterogeneous cell samples as compared to traditional bulk experiments. Moving from studying thousands of cells per sample into a few cells per sample gives better control and conditions to monitor live cells over time. In the presented microwell chip, the wells are small enough to fit 672 wells on the same area as a standard microscopic glass slide, a major advantage in clonal assays in terms of 672 parallel experiments. The small format is suitable both for non-fluorescent and fluorescently labeled cells, for example useful in fate mapping when examining various factors and their effect.

First and foremost, factors steering cells along certain differentiation paths are expensive. The reaction volume in the presented chip is 0.1% of the volume in a standard 96-well plate, meaning a 99.9% cost saving in reagent usage. Secondly, the high-density well format facilitates screening large numbers of cell samples simultaneously and thereby achieving a high throughput. Screening of 700,000 substances using this method would take less than two days, roughly estimated, using high-resolution data from one of the major bio-imaging systems. For lower resolution the corresponding time is three hours for 700,000 substances, using a conventional scanner system. Third, the transparency of the glass bottom and the satisfying optical properties of the chip enable high-resolution imaging.

Detection and imaging in the presented study was performed by manual microscopy in defined wells at certain time points. Automated imaging systems can be used for higher throughput and detection instruments validated so far in combination with the microwell chip are: Pathway (www.bdbiosciences.com), Cellavista (www.innovatis.com), Odyssey (www.licor.com), Starion (www.scienceimaging.se) and DNA Microarray Scanner (www.agilent.com). Manual pipetting, often with the aim of addressing many wells simultaneously was used for liquid handling in the present study. If different solutions are required in each well, an automatic dispensing unit is preferred. Examples of commercially available robotics that can efficiently dispense liquid onto the microwell chip are Equator (www.labcyte.com) and FlexDrop (www.perkinelmer.com). It should also be noted that due to the total volume of 500 nl per well, a single well can easily be targeted using a standard 0.5 µl pipette.

Proliferative heterogeneity within neural progenitor cell populations is well established [39]–[41] but the source of this variation is largely unknown. One influencing factor could be cell-cell contact and the impact of single-cell *vs.* high-density cell growth. Chin VI et al. demonstrated that the overall population growth was not enhanced by proximity to other cells, using a microfabricated array [15]. Nutrition uptake and cell behavior in limiting environments are other examples of experiments that should be well suited for the microwell chip presented here. Also, high-density microwell devices for successful screening of antigen-specific antibody-secreting cells have recently gained attraction, where different sizes (typically 50 µm in well-diameter) and number (typically >100,000 individual wells) of wells harbor single [42] or clonally expanded [43] antibody-secreting cells for a faster and more efficient cell-selection than using conventional methods.

A further understanding of adult and embryonic stem cells and their role in physiological and pathological conditions would increase the possibility of their use as potential therapeutics. Being able to study stem cells clonally in miniaturized devices could be useful in a wide range of screening applications. The presented microwell chip will probably find its most important use as an analysis platform. As for most *in vitro* assays, potential problems need to be taken into account. Obviously, passaging cells is more straightforward using conventional bulk procedures. The squared wells of the microwell chip could be a risk of increased cell positioning close to the wall, even though we have not detected such a pattern. Small wells can in some cases be difficult to handle, with regards to medium exchange, coating, or labeling. However, the present miniaturized format was proven to be at least as simple and fast as conventional larger formats.

Many assays that are run in 96-well plates today are promising applications for the microwell chip presented here, yielding a higher throughput, lower reagent consumption, and a better control of the cellular microenvironment. The possibility to perform PCR based genetic analysis on chip is currently under investigation, with promising results on correlation between cell culture and mutation frequency, all steps performed in individual wells on chip. Another example of research opening up for new applications would be controlled liquid handling by integrating microfluidics on this chip. In summary, several areas of use can be foreseen. Above all, the presented microwell chip should have high potential for applications like maintaining pluripotency, inducing reprogramming to the pluripotent state, and for screening of conditions leading to differentiation of stem cells into desired cell types.

To conclude, a chip-based platform for long-term differentiation studies on mouse and human ES cells and adult neural stem cells was demonstrated. Stem cells in microwell chips behave similarly to cells in conventional culturing systems, adding the advantages of small volumes, optimal imaging properties, controlled cell analysis and microenvironment, FACS compatibility, and a higher throughput. The presented microwell chip should simplify work-intensive and cost-demanding screening experiments by providing a wide range of applications within the field of stem cells, for example studying cell (de)-differentiation, reprogramming factors, cell heterogeneity, and cell-to-cell signaling.

## Materials and Methods

### Microwell chip design

Chip fabrication has previously been described by Lindström et al [37]. The outer format of the chip used in this study was that of a standard microscopic glass slide (26×76 mm^2^) with 672 wells. Each squared well has a bottom size of 650×650 µm^2^ resulting in a well volume of 500 nl. For cell culturing on chip a gas-permeable top membrane of polydimethylsiloxane (Sylgard 184, DowCorning, Midland, MI, http://www.dowcorning.com) was fabricated as described in detail elsewhere [37] with the only difference being the thickness of the membrane, in this study approximately 200 µm. Prior to cell culturing the chip and the top membrane were autoclaved.

### ES cell culture conditions

E14 ES cells derived from 129/Ola strain of mice [44] were maintained on 0.2% gelatin (Sigma-Aldrich, St Louis, MO, http://www.sigma-aldrich.com) coated dishes (Costar, Lowell, MA, http://www.corning.com) in Glasgow Minimum Essential Medium (Sigma) supplemented with 10% fetal calf serum (HyClone, Logan, UT, http://hyclone.com), 2 mM glutamax (Invitrogen, Carlsbad, CA, http://www.invitrogen.com), 1000 units of LIF, 1 mM sodium pyruvate, 10 µM non-essential amino acids (both from Invitrogen), 50 µM β-merchaptoethanol (Sigma) in 37°C, 5% CO_2_. The ES cells were passaged every second to third day using 0.05% Trypsin-EDTA (Invitrogen).

Human ES cell (HESC) lines used in this study were BG01 (BresaGen, Athens, GA, http://www.bresagen.com) and BG01V2. BG01V2 is a variant of BG01, characterized by trisomy of chromosome 17 [45]. The hESC were maintained in the undifferentiated state on mitomycin-C-treated mouse embryonic feeders (MEF) obtained from Millipore (Billerica, MA, http://www.millipore.com). Upon 80% confluency, hESC colonies were isolated from MEF layers by enzymatic treatment with 1 mg/ml collagenase type IV (Worthington Biochemical Corporation, Lakewood, NJ, http://www.worthington-biochem.com) for approximately 1 h, dissociated into small clusters, and re-plated on freshly prepared MEF layers. The cultures were maintained at 37°C, 5% CO_2_ in Dulbecco's Modified Eagle Medium (DMEM)/nutrient mixture, supplemented with 10% knockout serum replacement, 2 mM L-glutamine, 1 mM nonessential amino acid, 4 ng/ml bFGF, 50 U/ml Penn-Strep, and 0.1 mM ß-mercaptoethanol (All from Invitrogen).

### Neurosphere culture conditions

Neurosphere cultures were derived as described elsewhere [46] from adult C57bL/6 females. NS cultures were maintained at 37°C, 5% CO_2_ in uncoated plastic plates (Costar) in neurosphere medium containing DMEM/F12 medium supplemented with L-glutamine, B-27 (0.5 µg/ml), 20 ng/ml of both mouse bFGF (Peprotech, Rocky Hill, NJ, http://www.peprotech.com) and EGF (Peprotech) and Gentamycin (Invitrogen). NS cultures were passaged using 0.02% EDTA (Sigma) every third to fourth day.

### Pre-coating of the microwell chip

Before cell seeding, the microwell chip was pre-coated as described below. Gelatin (0.2%, Sigma) was used to coat the chip for ES cells, poly-L-lysine (0.01%, Sigma) was used for adult neural stem cells, and poly-L-ornithine (0.01%, Sigma) and laminin (20 ng/ml, Invitrogen) was used before hESC seeding. For coating with gelatin and poly-L-lysine, the required solution was added to the chip in a laminar airflow bench and incubated at room temperature for 15 min before emptying the wells through absorption on paper tissues (Precision Wipes, Kimtech Science, GA, http://www.kcprofessional.com) that had been UV-treated. For poly-L-ornithine and laminin coating, poly-L-ornithine was added to the chip and incubated for 10 min. The chip was washed with water and allowed to dry for approximately 30 min. Laminin was added to the chip which was incubated at 37°C for 20 min. The chip was washed 2× with phosphate buffered saline (PBS) before cells were introduced into the microwells.

### Stem cell seeding and culturing on chip

Stem cells were cultured on un-coated or pre-coated microwell chips using materials as described above. The cells were manually seeded into the microwells by random settling of the cells, in a concentration of 1×10^5^ cells/ml unless stated otherwise, resulting in approximately 50 cells per well. For automatic single-cell seeding into predefined microwells, a FACS Vantage SE Cell sorter (BD Biosciences, San Diego, CA, http://www.bdbiosciences.com) was employed, fitted with an x/y stage for plate sorting [37]. A top membrane was reversibly added to the chip to seal the wells, diminish evaporation, and contamination, followed by incubation at 37°C, 5% CO_2_. For long-term culturing and change of culture medium the top membrane was temporarily removed whereupon fresh medium was rinsed over the chip, displacing old medium in the microwells.

### Maintenance and differentiation of ES cells on chip

For mouse ES cells, two parallel experiments were performed in order to i) maintain ES pluripotency and ii) study neural ES cell differentiation. Two gelatin coated microwell chips were seeded with ES cells. On the first chip, culture medium change was performed on day 2, and fixation and labeling on day 3. The other chip was subjected to differentiation medium (specified below) on day 2, whereby medium was changed every third day until day 9 when the chip was fixed and subjected to staining. For induction of neural differentiation of mouse ES cells, the media was replaced with (DMEM)/F12 and neurobasal medium in a 1∶1 ratio, supplemented with 1% N2, 2% B-27, retinoic acid, human insulin, gentamycin (All from Invitrogen), and bovine serum albumin (Sigma).

For differentiation of human ES cells, clusters of BG01 or BG01V2 cells were resuspended in hESC culture medium without bFGF and transferred to ultra low-attachment plates (Corning Incorporated, NY, http://www.corning.com) for EB formation. After three days, the resulting EBs were dissociated into smaller cell aggregates composed of 100–150 cells and transferred to poly-L-ornithine/laminin coated microwell chips. To induce neural differentiation, the media was replaced with neurobasal medium supplemented with N2 and B-27.

### Neurosphere formation and neural differentiation of adult neural stem cells on chip

Three parallel experiments were performed; i) the first chip was left uncoated to promote free-floating NS formation, ii) the second chip was poly-L-lysine coated for plating and induction of neural differentiation (specified below) of adult neural stem cells, and iii) the third chip was poly-L-lysine coated for plating but maintained in NS medium supplemented with EGF and bFGF. Single-cell suspensions were prepared by passaging and filtering the cells (∅ 30 µm, BD Biosciences), followed by manual cell seeding. Wells containing i) a single cell or ii) several single-cells at experiment start were monitored during the experiments. Cell status on the three chips was imaged on day 3, 4, and the chip for neural differentiation (chip ii) was imaged on day 9. For induction of neural differentiation of adult neural stem cells, the media was replaced with DMEM/F12, supplemented with N2, B-27, and human insulin.

### Immunocytochemistry

Cells were fixed with 4% formaldehyde in PBS (Sigma) for 10 min, washed with PBS and incubated for 1 h with blocking solution, containing 10% Normal Donkey Serum (Jackson Lab, Bar Harbor, ME, http://www.jax.org/) in PBS and 0.2% Triton X-100 (Sigma). Cells were incubated for 40 min with the primary antibodies rabbit anti-Sox2 (1∶1000; Chemicon), mouse anti-βIII-tubulin (1∶1000; Covance, Princeton, NJ, http://www.covance.com), mouse anti-βIII-tubulin (1∶2000; Promega, Madison, WI, http://www.promega.com), mouse anti-Oct3/4 (1∶50; Santa Cruz, CA, http://www.scbt.com), mouse anti-Oct4 (1∶1000; Santa Cruz), anti-calreticulin, and anti-tubulin (1∶1000; both from Abcam, Cambridge, UK, http://www.abcam.com), anti-filamin antibody (1∶150, HPA000368, Stockholm, Sweden, http://www.proteinatlas.org). 2× washing was followed by 30 min incubation with secondary antibodies conjugated to either Cy3 (1∶500, Jackson Lab) or Alexa-488, -555, -647 (1∶1000, Molecular probes, Invitrogen). Cell nuclei were visualized with 4′-6-diamidino-2-phenylindole (DAPI, Sigma) at a dilution of 1∶5000 for about 1 min. Images were obtained using an Axioplan 2 imaging fluorescence microscope, Axiovert 200 M fluorescence microscope or one of the laser scanning confocal microscopes LSM 5 PASCAL or LSM 510 META (Carl Zeiss GmbH, Jena, Germany, http://www.zeiss.com) using a 10×, 20× or 40×W objective.
